# Medicines optimisation in Northern Ireland - scope and results of a challenging project

**DOI:** 10.1186/2052-3211-8-S1-P27

**Published:** 2015-10-05

**Authors:** Mike Scott, Frans van Andel, Rob Brenninkmeijer

**Affiliations:** 1 Optimisation Innovation Center, Belfast, BT4 3SQ, Northern Ireland; 2 Digitalis Mm Ltd, Dublin, 14, Ireland; 3 Digitalis BV, Amsterdam, 1075PW, Netherlands

## 

In Northern Ireland (NI), a Medicines Optimisation (MO) project has been successfully implemented by the Northern Health and Social Care Trust (NHSCT) over the last few years and continues to be developed.

Historically, the approach taken to reduce medicine expenditure has been to focus almost exclusively on costs and cost-cutting initiatives. This methodology has had only limited success, as it fails to address the more fundamental aspects of the quality and safety of medicine use. Hence, in NI a new strategy was adopted, based on the premise that quality and safety drive health gain and economy. Thus, the model STEPSelect was developed (Safe Therapeutic Economic Pharmaceutical Selection) to ensure that medicine selection is fundamentally based on clinically related content such as efficiency, safety, documented effects on clinical end points and ease of administration. STEPSelect is a web-based tool developed by Digitalis Mm Ltd, enabling clinicians and other health care providers and managers to comprehensively select and procure medicines and medical devices.

The rationale for STEPSelect is that successful introduction of new medicines does not merely depend on clinical features of medicines only. Other factors, which are decisive for early adoption of new medicines are the knowledge base clinicians have of new medicines relative to existing ones and ownership and fellow peer review, when deciding about prescribing new medicines. STEPSelect is based on System of Objectified Judgment Analysis (SOJA) technology. SOJA is a selection method to facilitate rational drug selection and create a new quality in prescribing based on objectified consensus among clinicians.

The STEPSelect method looks in the first instance at clinical features of health technologies. At a later stage of the evaluation, product quality and fitness for purpose are assessed (the so-called risk assessment stage) as well as the budget impact of a health technology and appropriate procurement steps and routines. Evaluations are carried out by Expert Groups, which are composed on the basis of a multidisciplinary nature consisting of clinicians, pharmacists, nurses and other staff as appropriate.

In NI the STEPSelect technology has been applied to procurement of medicines in many different therapeutic groups such as statins, erythropoietin stimulating agents (ESA's) and the use of biologicals in rheumatoid arthritis. Results with the method have invariably been positive in terms of support by clinicians and quality and cost reductions of prescribing, often in the region of 20-25% per therapeutic group.

